# Limb Amputation after Multiple Treatments of Tenosynovial Giant Cell Tumour: Series of 4 Dutch Cases

**DOI:** 10.1155/2017/7402570

**Published:** 2017-06-28

**Authors:** Monique J. L. Mastboom, Floortje G. M. Verspoor, Hans Gelderblom, Michiel A. J. van de Sande

**Affiliations:** ^1^Orthopaedic Surgery, Leiden University Medical Center, Leiden, Netherlands; ^2^Orthopaedic Surgery, Radboud University Medical Center, Nijmegen, Netherlands

## Abstract

In Tenosynovial Giant Cell Tumours (TGCT), previously named Pigmented Villonodular Synovitis (PVNS), a distinction is made between a single nodule (localized-type) and multiple nodules (diffuse-type). Diffuse-type is considered locally aggressive. Onset and extermination of this orphan disease remain unclear. Surgical resection is the most commonly performed treatment. Unfortunately, recurrences often occur (up to 92%), necessitating reoperations and adjuvant treatments. Once all treatments fail or if severe complications occur, limb amputation may become unavoidable. We describe four cases of above-knee amputation after TGCT diagnosis.

## 1. Background

Tenosynovial Giant Cell Tumour (TGCT) is considered an orphan, monoarticular, locally aggressive neoplasm [1]. TGCT patients complain of continued pain, swelling, and a decreased range of motion of the affected joint [2]. Typically, younger patients (below the age of 40 years) are affected. Time to definitive diagnosis usually takes several years [1]. TGCT develops along the synovial lining of joints, tendon sheaths, and bursae [1, 3]. Two extremes along a continuum of one disease process are described: a single nodule (localized-type) and multiple nodules (diffuse-type) [1, 2, 4]. These two subtypes differ in their clinical and radiological presentation, response to treatment, and prognosis. Histologically, no differences are detected [1, 5]. Exact onset remains unclear. Current findings are pleading for both a reactive inflammatory disorder and a clonal neoplastic proliferation, provoking a CSF1 overexpression, suggesting the tumour-landscaping effect [6]. The localized-type (Giant Cell Tumour of Tendon Sheath) is defined as a demarcated benign mass, most commonly occurring in fingers (85%). Lesions are small (between 0.5 and 4 cm), typically lobulated, and white to grey along with yellow and brown areas [1, 2, 4]. Reported recurrences ensuing surgical treatment are relatively low: 0–6% [4]. On the contrary, the diffuse-type (Diffuse-type Giant Cell Tumour (Dt-GCT), previously named Pigmented Villonodular Synovitis (PVNS)), shows extensive involvement of the entire synovial membrane and tends to have the capability to grow infiltrative through adjacent structures [2, 4]. Dt-GCT affects mostly weight-bearing joints: predominantly the knee joint (75%), followed by the hip-joint (15%). At present, surgery remains the gold standard, while systemic targeted treatments are only available in trial-settings [7]. Recurrence rates for Dt-GCT are 14% (up to 67) after open synovectomy and 40% (up to 92) after arthroscopic synovectomy [4]. Recurrent or resistant disease frequently necessitates multiple mutilating surgeries and ends occasionally inevitably in total joint arthroplasties [8]. Once all treatments fail or severe complications occur, limb amputation may become unavoidable. To our knowledge, current literature lacks reports of limb amputation in TGCT patients, but patient groups often discuss the possibility on online fora (“PVNS is pants” closed Facebook community; https://www.facebook.com/groups/91851410592/?ref=ts&fref=ts) [9]. To underline potential aggressiveness of TGCT, four patient history scenarios are described.

## 2. Case Presentation

### 2.1. Case  1

**Figure 1:**
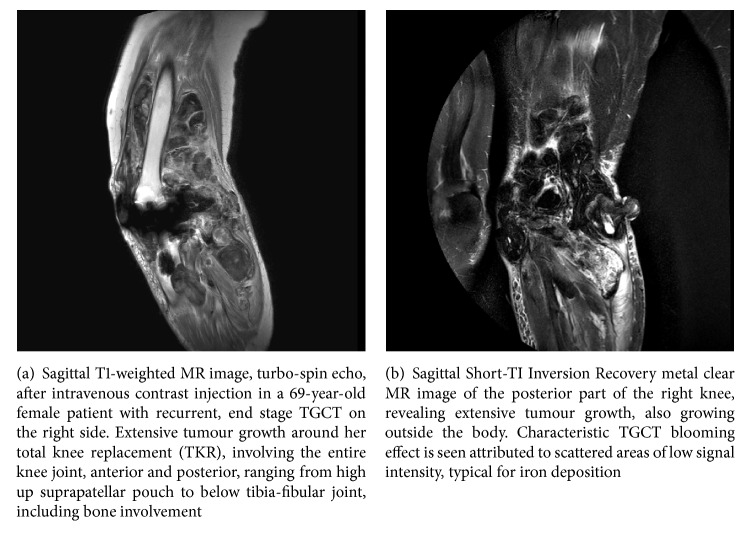
*Case  1*.

A female, aged 46, was diagnosed with TGCT. Initial TGCT treatment consisted of three arthroscopic synovectomies. First synovectomy was supplemented with low-dose radiation, consecutive two synovectomies with intra-articular 90Yttrium. Fourteen years later, a Magnetic Resonance Imaging (MRI) scan revealed recurrent TGCT, including bone involvement. A total knee replacement (TKR) was performed. Four years later, her knee started to hurt and swell again. Infection parameters were elevated, MRI showed extensive synovitis, and a PET-CT showed enhancement around her TKR, suspect for recurrent TGCT. Her range of motion was impaired, with a flexion-extension of 50-20-0. Twenty-three years after initial diagnosis, she was referred to our tertiary orthopaedic oncologic center. TGCT reexcision was not an option, as a result of extensive tumour growth (Figures 1(a) and 1(b)). Imatinib (a tyrosine-kinase-inhibitor with activity against CSF1R) was started for four months. Besides the tumour growing outwards from her operation scar, a nearby fistula was revealed and started leaking. She was admitted with malaise, fever, elevated infection parameters, a red swollen right leg, and not being able to mobilize. During four weeks of admission she was treated with several blood transfusions attributed to persistent anaemia, intravenous antibiotics, and analgesics. After an investigational tyrosine-kinase-inhibitor (TKI) in compassionate use was started, she was discharged. After a fall, a few days after she was discharged, her condition worsened. She was readmitted and treated with intravenous antibiotics for an acute Staphylococcus aureus infection, provoked by TGCT growing outside the operation scar composing a direct connection to the TKR. To avoid septic shock, an urgent above-knee amputation seemed like a live-saving procedure. Within one month, signs of osteomyelitis were revealed. Treatment with debridement, antibiotics, and irrigation stabilized the patient. At one-year follow-up, there were no signs of local recurrence or infection and her phantom pain was decreasing.

### 2.2. Case  2

**Figure 2:**
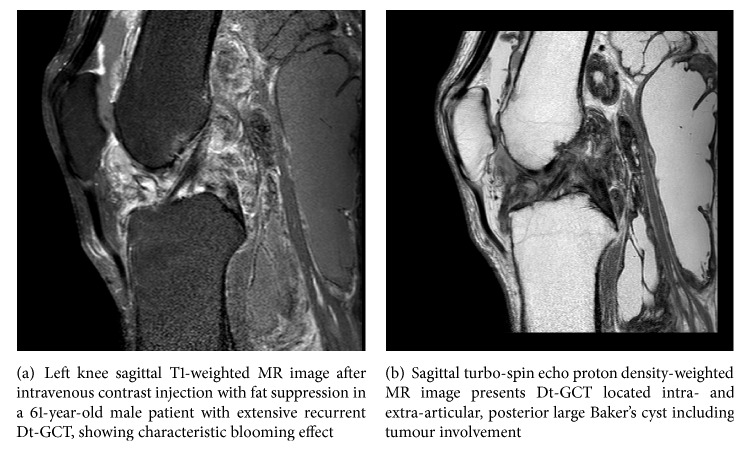
*Case  2*.

A 63-year-old male was referred to our tertiary hospital with recurrent Dt-GCT of his left knee. Two years prior to referral, Dt-GCT was diagnosed and (partial) arthroscopically removed elsewhere. MRI showed a diffuse-TGCT growth-pattern involving all compartments of the entire knee joint, including a Bakers cyst (Figures 2(a) and 2(b)). Consequently, a two-staged anterior and posterior synovectomy in two tempi was performed; macroscopically all pathological tissue was removed. There was chondromalacia grades 3-4. A few months later, the patient suffered progressive knee pain again. Recurrent Dt-GCT lesions, including bone involvement and progressive osteoarthritis, were seen on X-ray and MRI. A transarticular distal femoral resection and resection of all Dt-GCT tissue was performed. The knee joint was reconstructed using an endoprosthetic-reconstruction (EPR). Thereafter patient's knee function seemed to improve. However, several months later, swelling and increasing knee pain developed. C-reactive protein (CRP) and erythrocyte sedimentation rate were elevated; nevertheless cultures of aspirated knee fluid were negative. Along with general deterioration of the patient, wound debridement, antibiotics, irrigation, and retention (DAIR) was performed. Two out of six cultures, showed coagulase negative staphylococci without a sign of recurrent TGCT. Despite the DAIR procedure, his EPR had to be replaced with a gentamicin loaded spacer. Because of the difficulty to treat the low-grade infection, his spacer was replaced with a gentamicin and vancomycin loaded spacer. Thereafter, patient's condition improved, his infection parameters declined, and cultures of an open biopsy were negative. The EPR was reimplanted. Unfortunately the low-grade infection recurred again. After two additional DAIR procedures the patient preferred an above-knee amputation over another DAIR procedure, life-long antibiotics, or a third 2-stage revision. At present he is pain-free and ambulatory with an above-knee prosthetic leg.

### 2.3. Case  3

**Figure 3:**
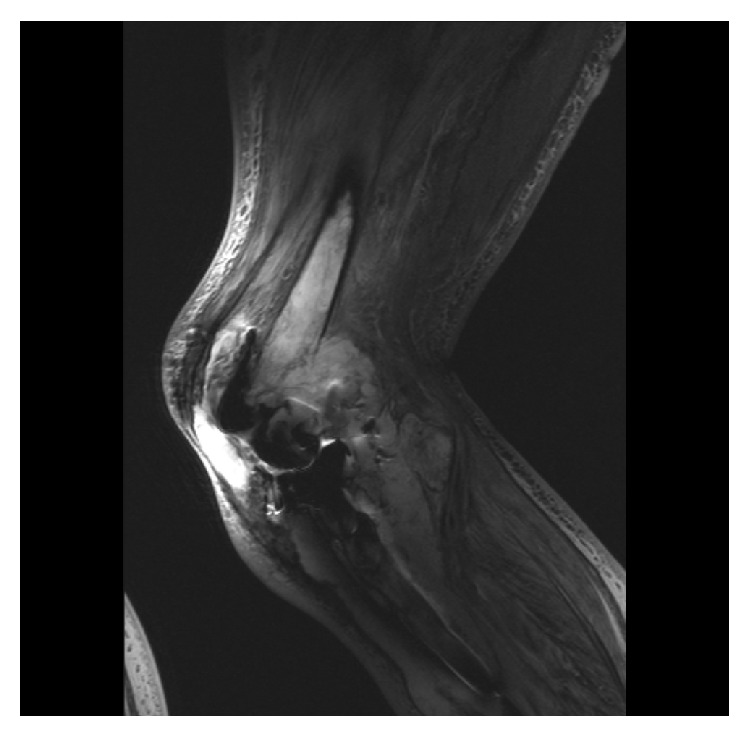
*Case  3*. Metal artefact reducing sequelae sagittal T2 weighted turbo inversion recovery MR image of the right knee of a 67-year-old male patient, with a TKR in situ. Extensive tumour progression around TKR and bone invasion is shown.

**Figure 4:**
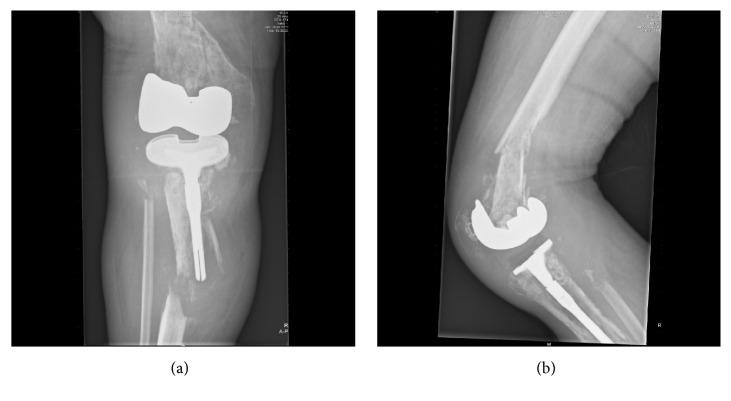
*Case  3*. X-rays (anterior-posterior and sagittal) of failing total knee replacement, attributed to aggressive TGCT progression including bone involvement, after radiotherapy treatment.

**Figure 5:**
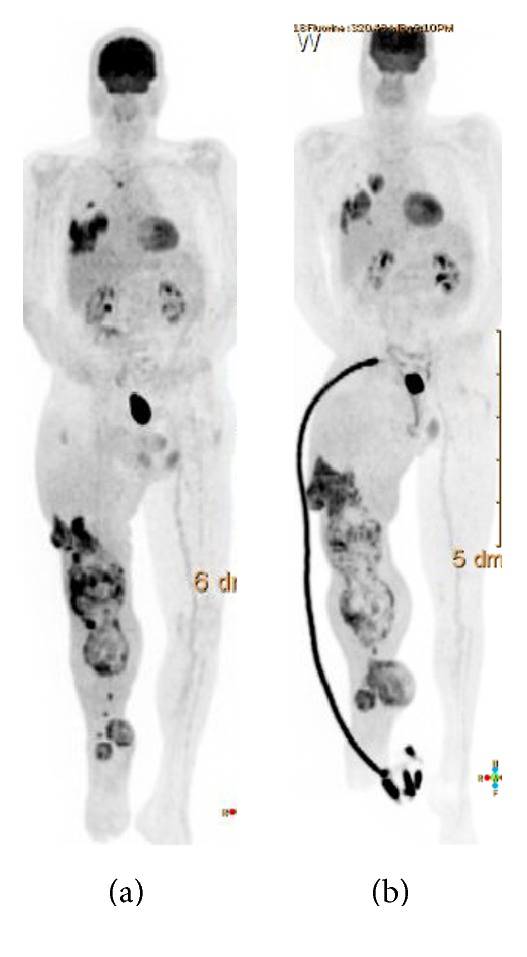
*Case  3*. PET-CT scan showing extensive TGCT around the right knee joint and multiple lung, pleural, and costal metastases. When pulmonary symptoms developed, an investigational tyrosine-kinase-inhibitor (TKI) was started ((a) prior to treatment; (b) after treatment), which had an effect on his pulmonary-metastases but not on his irradiated painful lymphedemic leg.

A 67-year-old male had a TKR after years of indistinct progressive knee pain. Peroperatively a benign tumour with few giant cells was diagnosed as a coincidental finding. A few months later a suprapatellar biopsy showed a mixed malignant appearance, including TGCT components. Unexpectedly, lymphadenopathy on his groin did not show malignant cells but reactive cells. The patient suffered of systemic symptoms: night sweats, weight loss, and infection-like symptoms (not specified). Both for the lymphadenopathy and his painful right knee he received radiotherapy (70 Gy on both locations, treatment for uncontrollable pain). Histopathologic revision, by a tertiary specialized pathologist in a reference center, showed a Dt-GCT. Aggressive tumour progression, including bone involvement, provoked TKR failure (Figures 3, 4(a), and 4(b)). Within one year, several histologically proven Dt-GCT lung metastases were discovered. Molecular research revealed a t(1;6)(p13;q27) translocation; this is not the typical t(1;2)(p13,q33) translocation; however literature shows different variants on this translocation. Final diagnosis through FISH technique confirmed Dt-GCT. Discomforting pulmonal symptoms expressed multiple lung, pleural, and costal metastases. Inside the thorax, numerous suspected lymph nodes were seen. When he developed pulmonary symptoms, an investigational TKI was started, which had an effect on his lung metastases but not on his irradiated painful lymphedemic leg (Figures 5(a) and 5(b)). Complaints of tiredness, disguise, a very oedematous right leg with a leaking protuberance, and persisting anaemia provided discomfort. Attributed to the TKI, pulmonary symptoms disappeared and his lung metastases stabilized. However, a hospital admission due to pneumonia on both sides and pulmonary embolisms caused a repercussion. As a last resort, the primary-tumour was resected by amputation, complicated with 4-litre blood loss and desaturation (until 90%), necessitating admission to the intensive care unit. Histopathology confirmed Dt-GCT without malignant cells; however margins were not disease-free. Residual and recurrent disease was seen on MRI three months postoperatively and clinically observed. After six months, a debulking procedure was performed on his amputated stump. The TKI did not show effect on the metastases anymore and was discontinued after one year of compassionate use. Currently, his phantom pain is acceptable.

### 2.4. Case  4

**Figure 6:**
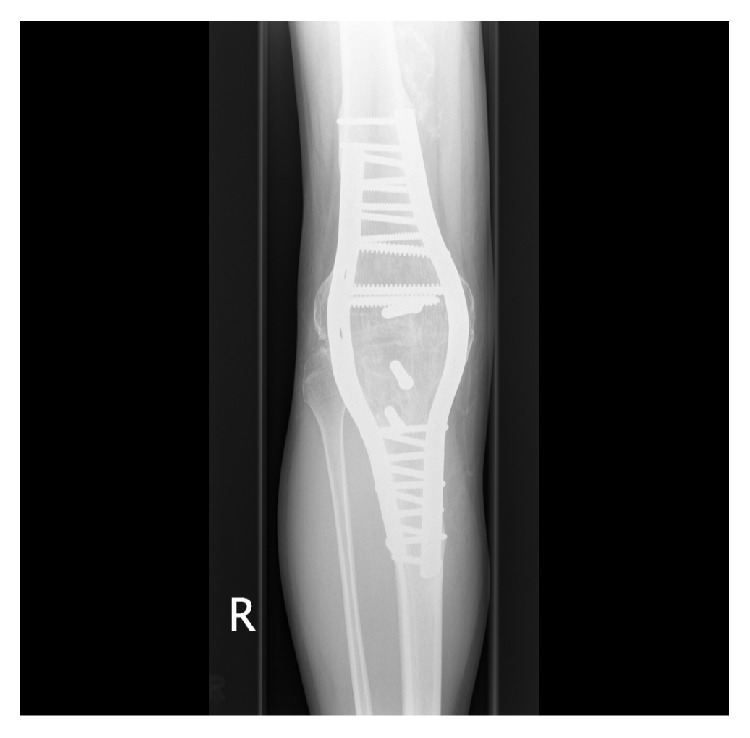
*Case  4*. Knee-arthrodesis after multiple Dt-GCT surgeries in a 26-year-old man, using a compression plate and screws.

**Figure 7:**
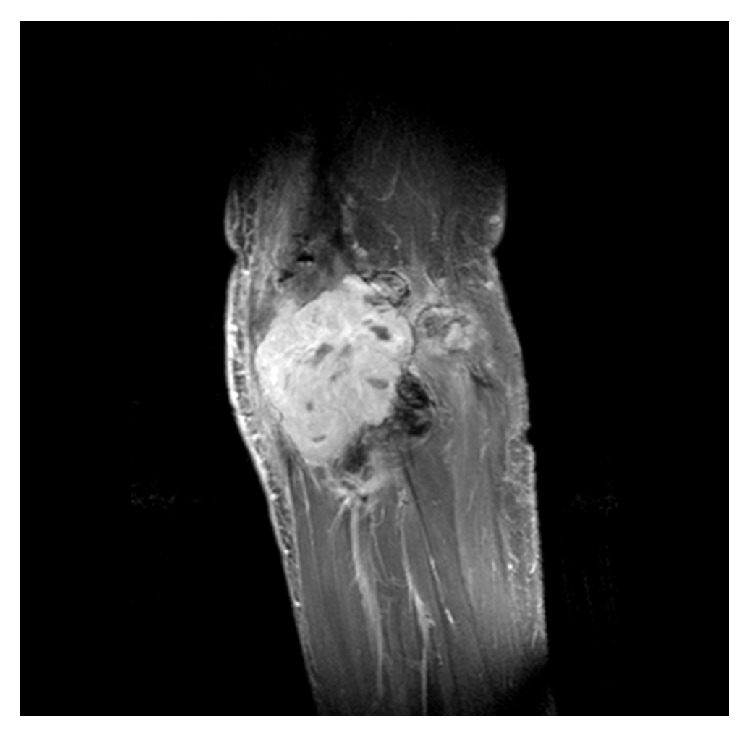
*Case  4*. Sagittal T1 weighted Spectral Presaturation with Inversion Recovery MR image after intravenous contrast, of a 28-year-old male patient revealing a large, extra-articular TGCT tumour mass. Patients history describes multiple surgical treatments, including removal of osteosynthesis material for a knee-arthrodesis.

After years of indistinct knee-complaints, a biopsy proved Dt-GCT in a 17-year-old male. Intra-articular 90Yttrium was not effective. After a partial open synovectomy, Dt-GCT recurred. A two-staged anterior and posterior synovectomy in two tempi (complicated by haemorrhage) was performed at a tertiary oncology center. During the following 13 years, the patient underwent a total of seven surgeries in an effort to treat Dt-GCT, including a knee-arthrodesis using a compression plate and screws (Figure 6). Osteosynthesis was removed several years later because of a low-grade osteomyelitis and persisting anaemia. Subsequently, a two-staged anterior and posterior debulking synovectomy was performed (Figure 7 shows MRI prior to debulking). After another debulking procedure, local tumour control did not seem feasible. An above-knee amputation was performed at the age of 30. Histopathological revision proved Dt-GCT, without malignant cells. After several years of painless walking with an external prosthesis, pulmonary symptoms occurred. Imatinib, an investigational TKI, chemotherapy, and radiotherapy had no effect on pulmonary and lymph node metastases. Despite all efforts, deterioration of the patient seemed irreversible. The patient deceased at the age of 35 years.

## 3. Discussion

TGCT onset is typically slow and patients present with unspecified complaints [1]. Pain, swelling, and stiffness in the involved joint might be misinterpreted as osteoarthritis, rheumatoid arthritis, a meniscal tear, or other ligamentous injury [2]. Because of the rarity of the disease, definitive diagnosis may take several years and patients present with extensive disease. Frequently, patients are referred to a tertiary hospital, after several arthroscopic or open synovectomies and even radiotherapy (Case number 1) [10, 11]. Besides declined functional outcome and quality of life [10], these patients are at risk of repeated recurrences and extensive resistant disease [7]. Multiple surgeries increase the risk of infection. Continued inflammation, joint usuration, and bone involvement may lead to articular destruction that might worsen (preexisting) osteoarthritis [2]. A total joint replacement or even an endoprosthetic-reconstruction may become inevitable [8, 12]. Occasionally, total joint arthroplasty is the primary procedure performed in TGCT [8]. Only rarely, an above-knee amputation as a last resort in treatment of TGCT is mentioned [13–16].

Is an above-knee amputation justified in an essentially benign, but locally aggressive disease? After (major) complications, for example, periprosthetic infections, in primary total knee arthroplasties, above-knee amputations are performed [17, 18]. Our amputation cases are also attributed to severe prosthetic infections (Cases  1 and 2). Radiotherapy, applied in Cases  1 (in a nonspecialized hospital) and 3 (in order to decrease severe pain-complaints), increases risk of prosthetic failure, infection, and wound healing. The overall prevalence of above-the-knee amputation after TKA is estimated at 0.36% [17]. When severe pain, a swollen joint, limited range of motion, and stiffness impair range of motion, an above-knee amputation might increase patients mobility [17, 19]. Therefore, we feel amputation is justified in extreme TGCT cases.

TGCT is a heterogeneous disease. Some cases are instantly diffusely spread intra- and extra-articularly or even show malignant characteristics. Metastases in histologically benign TGCT are extremely rare, called an implantation phenomenon, and conservatively treatment is suggested [14]. Symptomatic-free metastases in Case  3 were conservatively treated. Physicians should be aware of the potentially aggressive course of TGCT. Multiple mutilating surgeries decline functional outcome and quality of life [10]. Expert centers need to cooperate on these rare cases to understand the biology underlying these different clinical behaviours.

West et al. discovered a central role for CSF1 in the pathogenesis of TGCT [6]. Multiple trials with systemic therapies targeting CSF-1 receptor show promising results as novel treatment method for diffuse-TGCT [7]. Emactuzumab (RG7155) (a monoclonal antibody against CSF1R) showed an objective response in 26 of 28 (86%) TGCT patients [20]. Prolonged tumour regression is described in patients, treated with tyrosine-kinase-inhibitor PLX3397 [21]. (Serious) adverse events in emactuzumab and PLX3397 are investigated. Currently, two studies are recruiting patients with recurrent or unresectable TGCT diffuse-type: MCS110 (a CSF1-directed monoclonal antibody, NCT01643850) and FPA008 (an anti-CSF1R monoclonal antibody, NCT02471716). In the near future, if these systemic treatments are approved, multiple surgeries and final limb amputation, hopefully, will become obsolete.

To our knowledge, this is the first case-series focusing on limb amputation after multiple treatments of TGCT. In order to prevent extensive final treatments, like amputations, further investigation of TGCT risk factors for recurrences is essential in proper primary-treatment planning. In the orphan TGCT, knowledge of disease impact can be improved. Patients suffering extensive disease including patients after multiple mutilating surgeries might experience higher quality of life once they feel in control of their own life again. Performing an above-knee amputation may therefore be considered in extreme and extensive TGCT cases.

## 4. Conclusion

Frequently, TGCT is successfully treated with radical surgical excision. In a substantial percentage of cases, it presents as an aggressive and extensive disease that requires complex treatments and, in extreme cases, can even lead to limb-sacrificing surgery. Quick diagnosis and adequate treatment of this rare condition are important factors for outcome. Therefore, it is essential that these patients get referred to specialized centers at an early stage. We described four extensive Dt-GCT cases, treated with an above-knee amputation as final treatment.

## Additional Points

*Learning Points/Take Home Message*. (i) Consider TGCT in unspecified complaints: pain, swelling, limited range of motion, and stiffness in the involved joint. (ii) TGCT in an earlier, less extensive stage might be easier to cure. (iii) Radical surgery, including limb amputation, should be considered and discussed with the patient, for cases of recurrent and/or resistant TGCT. (iv) Systemic therapies targeting CSF-1 receptor show promising results as novel treatment method for diffuse-TGCT.
